# Neutrophil Activation and Immune Thrombosis Profiles Persist in Convalescent COVID-19

**DOI:** 10.1007/s10875-023-01459-x

**Published:** 2023-03-21

**Authors:** Hakim Hocini, Aurélie Wiedemann, Fabiola Blengio, Cécile Lefebvre, Minerva Cervantes-Gonzalez, Emile Foucat, Pascaline Tisserand, Mathieu Surenaud, Séverin Coléon, Mélanie Prague, Lydia Guillaumat, Corinne Krief, Craig Fenwick, Cédric Laouénan, Lila Bouadma, Jade Ghosn, Giuseppe Pantaleo, Rodolphe Thiébaut, the French COVID cohort study group, Laurent Abel, Amal Abrous, Claire Andrejak, François Angoulvant, Delphine Bachelet, Marie Bartoli, Sylvie Behilill, Marine Beluze, Krishna Bhavsar, Anissa Chair, Charlotte Charpentier, Léo Chenard, Catherine Chirouze, Sandrine Couffin-cadiergues, Camille Couffignal, Nathalie DE. Castro, Marie-Pierre Debray, Dominique Deplanque, Diane Descamps, Alpha Diallo, Fernanda Dias DA Silva, Céline Dorival, Xavier Duval, Philippine Eloy, Vincent Enouf, Hélène Esperou, Marina Esposito-farese, Manuel Etienne, Aline-Marie Florence, Alexandre Gaymard, Tristan Gigante, Morgane Gilg, François Goehringer, Jérémie Guedj, Ikram Houas, Isabelle Hoffmann, Jean-Sébastien Hulot, Salma Jaafoura, Simon Jamard, Ouifiya Kafif, Antoine Khalil, Nadhem Lafhej, Samira Laribi, Minh Le, Quentin LE. Hingrat, Soizic LE. Mestre, Sophie Letrou, Bruno Lina, Guillaume Lingas, Denis Malvy, France Mentré, Hugo Mouquet, Nadège Neant, Christelle Paul, Aurélie Papadopoulos, Ventzislava Petrov-sanchez, Gilles Peytavin, Valentine Piquard, Olivier Picone, Manuel Rosa-calatrava, Bénédicte Rossignol, Patrick Rossignol, Carine Roy, Marion Schneider, Coralie Tardivon, Jean-François Timsit, Sarah Tubiana, Sylvie VAN. DER. Werf, Benoit Visseaux, Yves Lévy

**Affiliations:** 1 Vaccine Research Institute, Université Paris-Est Créteil, Faculté de Médecine, INSERM U955, Team 16, Créteil, France; 2 Department of Public Health, Univ. Bordeaux, Inserm Bordeaux Population Health Research Centre, Inria SISTM, UMR 1219, Bordeaux, France; 3 Service of Immunology and Allergy, Department of Medicine, Lausanne University Hospital and University of Lausanne, Lausanne, Switzerland; 4 Département Épidémiologie Biostatistiques Et Recherche Clinique, AP-HP, Hôpital Bichat, INSERM, Centre d’Investigation Clinique-Epidémiologie Clinique 1425, 75018 Paris, France; 5 UMR 1137, Université de Paris, INSERM, IAME, 75018 Paris, France; 6 APHP- Hôpital Bichat – Médecine Intensive et Réanimation des Maladies Infectieuses, Paris, France; 7 AP-HP, Hôpital Bichat, Service de Maladies Infectieuses Et Tropicales, 75018 Paris, France; 8 Swiss Vaccine Research Institute, Lausanne University Hospital, University of Lausanne, Lausanne, Switzerland; 9 CHU de Bordeaux, Pôle de Santé Publique, Service d’Information Médicale, Bordeaux, France; 10 Assistance Publique-Hôpitaux de Paris, Service Immunologie Clinique, Groupe Henri-Mondor Albert-Chenevier, Créteil, France

**Keywords:** COVID-19 disease, post-acute COVID-19 syndrome, thrombosis

## Abstract

**Purpose:**

Following a severe COVID-19 infection, a proportion of individuals develop prolonged symptoms. We investigated the immunological dysfunction that underlies the persistence of symptoms months after the resolution of acute COVID-19.

**Methods:**

We analyzed cytokines, cell phenotypes, SARS-CoV-2 spike-specific and neutralizing antibodies, and whole blood gene expression profiles in convalescent severe COVID-19 patients 1, 3, and 6 months following hospital discharge.

**Results:**

We observed persistent abnormalities until month 6 marked by (i) high serum levels of monocyte/macrophage and endothelial activation markers, chemotaxis, and hematopoietic cytokines; (ii) a high frequency of central memory CD4^+^ and effector CD8^+^ T cells; (iii) a decrease in anti-SARS-CoV-2 spike and neutralizing antibodies; and (iv) an upregulation of genes related to platelet, neutrophil activation, erythrocytes, myeloid cell differentiation, and RUNX1 signaling. We identified a “core gene signature” associated with a history of thrombotic events, with upregulation of a set of genes involved in neutrophil activation, platelet, hematopoiesis, and blood coagulation.

**Conclusion:**

The lack of restoration of gene expression to a normal profile after up to 6 months of follow-up, even in asymptomatic patients who experienced severe COVID-19, signals the need to carefully extend their clinical follow-up and propose preventive measures.

**Supplementary Information:**

The online version contains supplementary material available at 10.1007/s10875-023-01459-x.

## Introduction

Following a severe acute respiratory syndrome coronavirus 2 (SARS-CoV-2) infection, a significant proportion of individuals develop prolonged symptoms, including predominantly fatigue, headache, and upper respiratory and multi-system complaints, including fever and gastroenterological symptoms [1]. Several studies have investigated immune and inflammatory dysregulation in acute and severe COVID-19 with the aim of identifying biomarkers of severity or predictive of clinical outcomes. However, the exact mechanisms behind the persistence of symptoms are yet to be identified but they probably result in the COVID-19 sequelae of organ damage, persistence of chronic inflammation, and dysregulation of the immune system [1]. Dysregulation of immune responses, including T-cell lymphopenia and exhaustion [2], and elevated serum levels of pro-inflammatory cytokines or alarmins are associated with a severe prognosis [3, 4]. In addition, we and others have reported a crucial role for neutrophil activation in the pathology of severe COVID-19 through the upregulation of multiple genes involved in their activation and migration [3, 5, 6]. A role for neutrophil extracellular traps (NETosis), both in COVID-19 acute respiratory distress syndrome (ARDS) and thrombotic events, has also been observed [7–9]. Concomitantly, severe COVID-19 has also been shown to be associated with altered hematopoiesis, as shown by lymphopenia and an increase in myelopoiesis [10, 11]. Single-cell analysis of bone marrow mononuclear cells from severe COVID-19 patients showed an accumulation of immature myeloid and a reduction of lymphoid progenitors, along with the upregulation of transcription factors (TF) regulating the differentiation of hematopoietic stem cells into downstream progenitors [12].

Concerning the adaptive immune response to SARS-CoV-2, most COVID-19 patients develop antibodies and T-cell to SARS-CoV-2 antigens [13–15]. In the post-acute phase, high titers of specific immunoglobulin G and neutralizing antibodies are detected [16, 17], as well as specific and polyfunctional T cells with an early differentiated memory phenotype associated with stem-like properties [14, 18, 19]. These observations underscore the complexity of the pathophysiological process underlying acute and severe COVID-19. The duration of such dysregulation following the acute phase of the infection is yet to be investigated. In this context, we investigated immune cell phenotypes, SARS-CoV-2 spike specific and neutralizing antibodies, serum biomarkers, and whole blood gene expression profiles in a cohort of hospitalized COVID-19 patients followed from acute phase infection through convalescence 1, 3, and 6 months following discharge from the hospital.

## Methods

### *Participants*

We enrolled a subgroup of COVID-19 patients of the prospective French COVID cohort (registered at clinicaltrials.gov NCT04262921). Eligible patients were those who were hospitalized with virologically confirmed COVID-19 by PCR performed on the day of inclusion*.* The definition criteria of the severity of the disease were stated according to WHO and French National Health Agency guidelines applied to the inclusion of the French National COVID-19 cohort “French cohort” [20]. Viral loads were quantified by real-time semi-quantitative reverse transcriptase polymerase chain reactions (RT-PCR) using either the Charité WHO protocol (testing the E gene and RdRp) or the Pasteur institute assay (testing the E gene and two other RdRp targets, IP2 and IP4). Convalescent follow-up visits were performed at 2 to 4 weeks after discharge (month 1), and/or month 3, and/or month 6. Among convalescent patients included in the transcriptomic study, 43% and 41% were symptomatic at M3 and M6, respectively. The study was conducted with the understanding and consent of each participant or their surrogate covering the sampling, storage, and use of biological samples. The healthy donors (HD) were sampled before COVID-19 outbreak, their characteristics are shown in Table S3.

### Quantification of Serum Biomarkers, Anti-SARS-CoV-2 Spike Antibody, and Pseudo-Neutralization Assay.

We quantified 29 biomarkers in serum samples. A Human XL Cyt Disc Premixed Mag Luminex® Perf Assay Kit (R&D Systems) was used to measure the following 22 biomarkers: CCL2/MCP-1, CCL4/MIP-1β, RANTES/CCL5, CCL11/Eotaxin, CCL19/MIP-3β, CCL20/MIP-3α, CD40Ligand, Fractalkine/CX3CL1, CXCL10/IP-10, EGF, Flt-3 Ligand, Granzyme B, IL-1ra, IL-6, IL-7, IL-8/CXCL8, IL-10, IL-15, PD-L1/B7-H1, TNF-α, TRAIL/TNFSF10, and VEGF. CD163, ST2, CD14, and LBP were measured with a Human Magnetic Luminex® Assay (R&D Systems). Luminex assays were performed according to the manufacturer’s recommendations and read on a Bio-Plex 200 system™ (Bio-Rad). Amphiregulin, FABP2/IFABP, and haptoglobin were measured using Human Quantikine ELISA Kits (R&D Systems) according to the manufacturer’s instructions. Anti-SARS-CoV-2 spike immunoglobulin G (IgG) and neutralization levels were determined as already reported [21, 22] and detailed in the supplementary data.

### RNA Extraction, Library Preparation, and Sequencing.

Gene expression profiling was carried out on the whole blood of 10 HD and of 22, 25, and 18 samples of convalescent patients. Eight, 12, and 9 convalescent patients were sampled only at M1, M3, and M6, respectively. In contrast, 8 patients were sampled both at M1 and M3, 4 patients at M1 and M6, 3 patients at M3 and M6, and only 2 patients at M1, M3, and M6. Total RNA was purified using the Tempus™ Spin RNA Isolation Kit (ThermoFisher Scientific). RNA was quantified using the Quant-iT RiboGreen RNA Assay Kit (Thermo Fisher Scientific) and quality control performed on a Bioanalyzer (Agilent). Globin mRNA was depleted using the Globinclear Kit (Invitrogen) prior to mRNA library preparation with the TruSeq® Stranded mRNA kit, according to the Illumina protocol. Libraries were sequenced using an Illumina HiSeq 2500 V4 system.

### Differential Gene Expression Analysis

Sequencing quality control was performed using Sequence Analysis Viewer and FastQ files were generated on the Illumina BaseSpace Hub. After trimming (QPhred score ≥ 25) with Bowtie 2–2.2.5 software, reads were aligned with the hg19 human reference genome using STAR-2.5.3ar and quantified relative to annotation model hg19—GENCODE Genes—release 19 using Partek E/M (Partek® Flow® software, v10.0). Differential gene expression analysis was performed using the DearSeq package [23]. Differentially expressed genes (DEGs) with adjusted *p* values ≤ 0.05 and a fold-change in expression ≥ Log2 0.58 were subjected to functional enrichment analysis using Metascape software with the default parameters (https://metascape.org) [24]. Geom_line and geom smooth functions from ggplot2 [25] were used to generate line plots for the gene trend visualization using the LOESS smoothing method, which fits a polynomial surface determined by one or more numerical predictors using local fitting.

### Construction of the Protein–Protein Interaction (PPI) Network and Molecular Complex Detection (MCODE)

The interaction network among proteins encoded by the DEGs was established by importing the selected genes into the STRING database v11.5 [26], (http://string-db.org). To remove PPI that were inconsistent from the dataset, we used the standard cut-off of a confident interaction score ≥ 0.4. Cytoscape software version 3.9.0 [27] was applied for the genes network visualization. The MCODE [28] plug-in of the Cytoscape tool was used to visualize the significant gene modules in each network, by default, with a cut-off k-core = 3 based on the MCODE analysis.

### Clustering of Convalescent Severe COVID-19 Patients and Healthy Donors

Hierarchical clustering was used to observe gene expression patterns between subjects and for cluster identification. Feature selection was based on the most highly variable genes (IQR > 0.7). The R package clValid [29] was used to identify and validate the cluster algorithm (Figure S7). The “average silhouette width” index from the factoextra R package [30] was used to determine the optimal number of clusters. The fviz dend function from the factoextra package was then used to draw the dendrogram.

### Statistics

Graphpad Prism software version 8 was used for nonparametric statistics and plots as described in the figure legends. Heatmaps were generated using the function aheatmap from the NMF package in R software, version 4.0.0. R: A language and environment for statistical computing. R Foundation for Statistical Computing, Vienna, Austria. URL: https://www.R-project.org/. Differences in the expression of standardized biomarkers were analyzed using nonparametric Wilcoxon tests, adjusting for multiple testing using the Benjamini & Hochberg correction. A leave-one-out procedure was performed to assess the robustness of the result regarding outlier patients. We performed the same analyses in turn in all but one of the subjects of the dataset and assessed if it impacted which analytes are found to be differently expressed between COVID-19 patients and health donors at each time points.

## Results

### Participants

We recruited 100 hospitalized COVID-19 patients from the French COVID cohort [20]. The median (IQR) age was 59 (47–67) years, 66% were male, and 81% were hospitalized in intensive care units (ICUs) during the acute phase of infection. At month (M) 3 and M6 post-hospitalization, 28% and 45% of the patients had three or more persistent symptoms, respectively. Fatigue, abnormal pulmonary auscultation, and dyspnea were the most frequent symptoms. Whole blood samples were obtained longitudinally (2–3 time points) from 33 patients or at a single time point from 69 patients. The demographic and clinical characteristics of patients and the details of the blood samples used in each assay are summarized in Tables S1 and S2.

### Overview of Serum Biomarkers and Cellular and Humoral Characteristics of Convalescent COVID-19 Patients

**Fig. 1:**
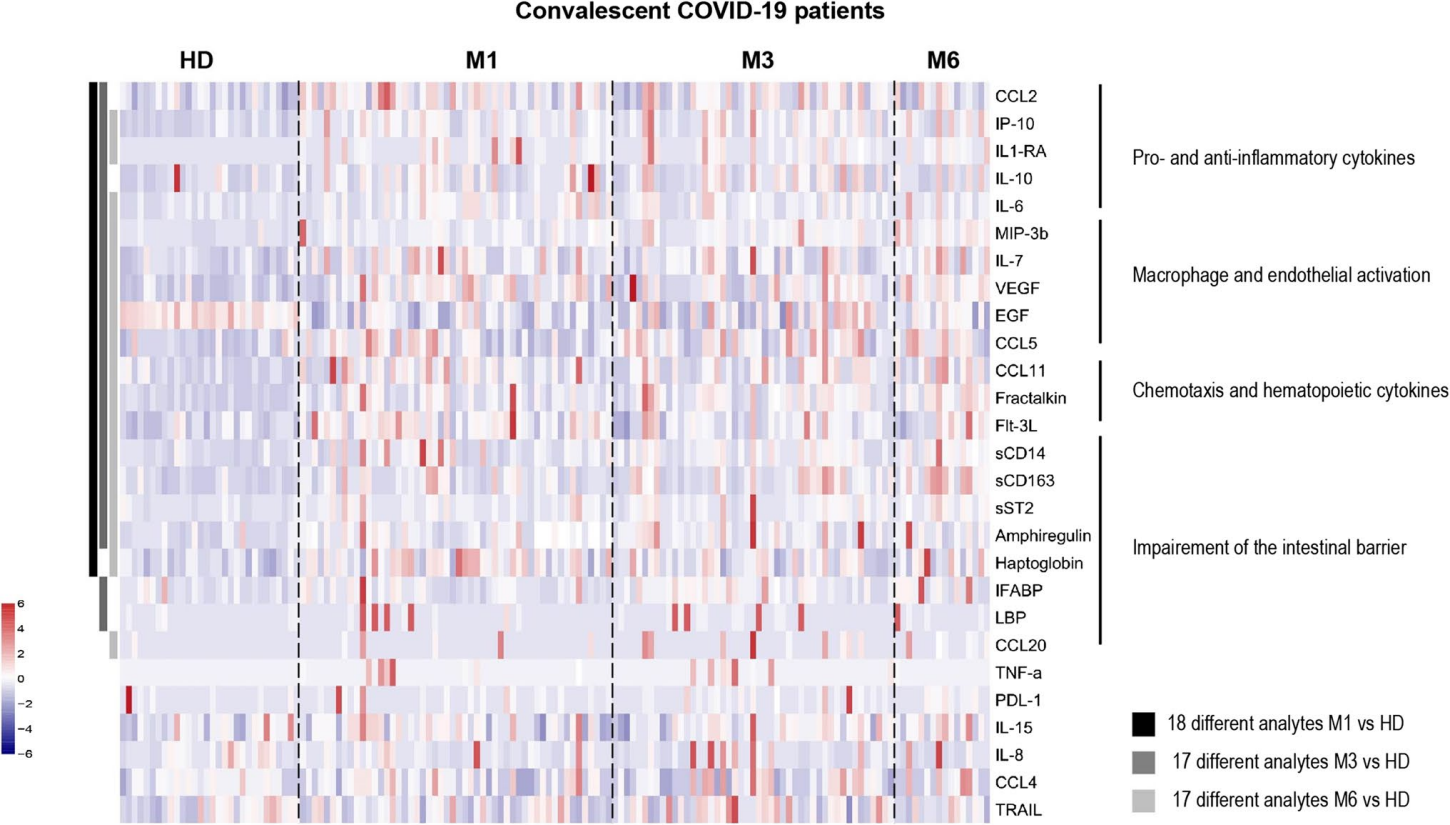
Heatmap of standardized biomarker expression in the serum of convalescent COVID-19 patients at M1, M3, and M6 post-infection. The colors represent standardized expression values centered on the mean, with variance equal to 1. Biomarker hierarchical clustering was computed using the Euclidean distance and Ward’s method. HD, healthy donors (*n* = 30); M1 (*n* = 42), M3 (*n* = 47), M6 (*n* = 16). The vertical lines on the left side of the heatmap represent which analytes levels are different between healthy donors and covid19 patients at M1 (left, dark-grey), M3 (middle, middle-grey), and M6 (right, light-grey) time points

The levels of 21 biomarkers differed significantly in convalescent COVID-19 patients compared to healthy donors (HD, *n* = 30) (Wilcoxon test adjusted for multiple comparisons) (Fig. 1 and Figure S1) at various time points, of which most persisted until M6 following the acute phase of infection. Among the biomarkers showing differences, the levels of pro-inflammatory factors (CCL2, IP-10, and IL-6) and anti-inflammatory cytokines (IL-1-RA and IL-10) were elevated at M1 and M3. The levels of these factors, but not those of CCL2 or IL-10, were still high at M6. Macrophage and endothelial activation marker (MIP-3β, IL-7, VEGF, EGF, and CCL5) levels were also elevated up through M6. Accordingly, elevated levels of soluble CD163 (sCD163), a specific marker of monocyte/macrophage activation, and chemotaxis and hematopoietic cytokines (CCL11, fractalkine, and Flt-3L) also persisted up to 6 months. The levels of lipopolysaccharide-binding protein (LBP) and intestinal fatty acid-binding protein (iFABP), a marker of gut epithelial damage, were also significantly elevated in convalescent COVID-19 patients at M3. Consistent with this finding, the levels of markers of intestinal epithelial regeneration (EGF, amphiregulin AREG, and haptoglobin), soluble ST2, and sCD14, a marker of intestinal translocation, were elevated at various time points up to M6 (Fig. 1 and Figure S1E). Using a leave-one-out validation, we demonstrated that these findings are robust to outliers patients.

We also performed phenotypic analysis of the blood immune cells from the convalescent COVID-19 patients. There was a significant reduction in the frequency of total CD3^+^ T cells up to 3 months after the acute phase relative to HD (*P* = 0.099 and *P* = 0.0375 at M1 and M3, respectively), whereas the number of central memory CD4^+^ T cells was elevated at M1 and M3 (*P* = 0.0073 and *P* = 0.0143, respectively). The frequency of naïve CD8^+^ T cells was reduced at M1, whereas the frequency of effector memory CD8^+^ T cells was elevated at M1 and M3 and they expressed an activated cell phenotype (CD38^+^HLA-DR^+^) until M1 (*P* < 0.0001 relative to HD) (Figure S2A).

The mean fluorescence intensity (MFI) of anti-SARS-CoV-2 spike antibody for convalescent COVID-19 patients was 368.8 (252.2–610.8) fold higher at M1 than that for a pool of HD, which then decreased by M3 (201.7 (142.7–342.1)) and M6 (176.5 (135–258)) (*P* < 0.0001, for all time points) (Figure S2B). Similarly, the percentage of inhibition of these antibodies was 68.6 (58–84) at M1, which decreased to 37.6 (29–57) and 41.9 (33–50) by M3 and M6, respectively (Figure S2B).

### Changes in Whole Blood Gene Expression in Convalescent COVID-19 Patients Relative to HD

**Fig. 2:**
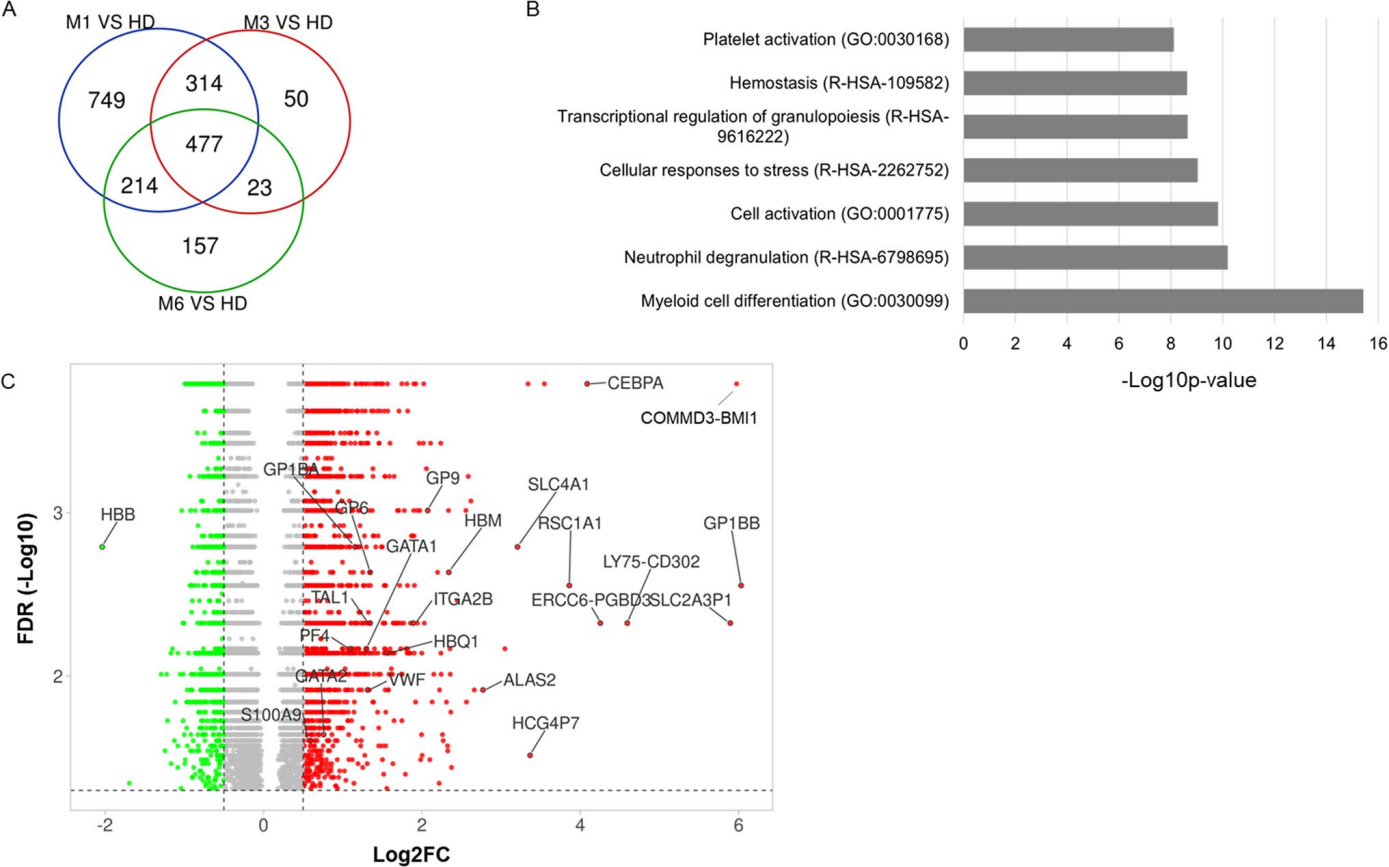
Differentially expressed genes between convalescent patients at M1, M3, and M6 relative to healthy donors (HD). **A** Venn diagram of the number of DEGs between convalescent patients at M1, M3, and M6 relative to HD. **B** Pathways associated with DEGs between convalescent at M1 and HD. **C** Volcano plot of DEGs with an FDR ≤ 0.05 between convalescent COVID-19 patients at M1 relative to HD. Up- and downregulated genes are represented by red and green dots, respectively

To characterize the transcriptomic changes that occur in convalescent COVID-19 patients, we investigated DEGs between samples collected at M1 (*n* = 22), M3 (*n* = 25), and M6 (*n* = 18) and HD (*n* = 10). We identified 1754, 864, and 871 DEGs at M1, M3, and M6, respectively (Fig, 2A). The DEGs at M1 involved pathways of myeloid cell differentiation, neutrophil degranulation, transcriptional regulation of granulocytes, and platelet activation (Fig. 2B). Among the top upregulated genes, some are involved in myeloid lineage and required for granulocyte formation (*CEBPA* and *GATA1*). Others are involved in erythrocyte and platelet differentiation and activation, such as *ALAS2*, *GP1BB*, GP6, GP9, *VWF*, *GP1BA*, and *HBQ1*, which is normally found in human fetal erythroid tissue. Interestingly, the top downregulated gene, *HBB*, is one of the subunits of adult hemoglobin (Fig. 2C). Aside from these genes, at M1 after hospital discharge, we also observed dysregulation of genes related to the myeloid lineage (Figure S3A), which persisted until M6 (Figure S3B). PPI analysis (Figure S3C) identified the main network, which involved the upregulation of TF genes associated with myeloid and erythrocyte cell differentiation (*CEBPA*, *CEBPE*, *GATA1*, *GATA2*, and *KLF1*), as well as genes belonging to GP1b-IX-V activation (*GP1BB*, *GP9*, and *GP1BA*). Of note, the GP1b-IX-V complex is a platelet receptor that mediates the initial interaction with subendothelial vWF which levels were elevated in COVID-19 patients [31]. The involvement of platelet activation was confirmed, as shown by the dysregulation of multiple genes related to platelets (Figure S4A). Among these genes, we observed two main clusters. The first cluster (C1) consisted of the genes downregulated at M1 relative to HD (*PRKACB*, *ENPP4*, *HBB*, and *FZD6*) (Figure S4A), which are involved in blood coagulation. The second cluster (C2) included genes upregulated at M1 that are mainly involved in F9 and GP1b-IX-V activation, platelet adhesion and aggregation, and plug formation. All these genes were dysregulated at M1 relative to HD and did not return to HD level at M6 (Figure S4B). The PPI results confirm the key role of the blood coagulation and platelet activation in the dysregulated mechanisms observed at day 1 (Figure S4C).

We have previously reported that changes in neutrophil activation genes are predictive of severe disease in COVID-19 [5]. Thus, we took the opportunity of the longitudinal follow-up of cohort of convalescent COVID-19 patients to follow neutrophil gene pathways. We observed modified expression of many genes associated with neutrophil activation (Figure S5A). Two main clusters of DEGs were identified that exhibited different dynamics. One cluster decreased expression at M1 and remained downregulated, despite a tendency to increase by M6. By contrast, the expression of genes in the second cluster was increased at M1 and remained higher up to M6 (Figure S5B). This cluster included genes of neutrophil degranulation (*S100A9, DEFA1B, A1BG, MMP9, PPBP, SIGLEC5,* and *SIGLEC9*). Network analysis of the neutrophil genes identified major pathways concerning neutrophil activation/degranulation and complement C3b/C4b receptor 1 and C5a receptor 1 (C5AR1) (Figure S5C).

**Fig. 3:**
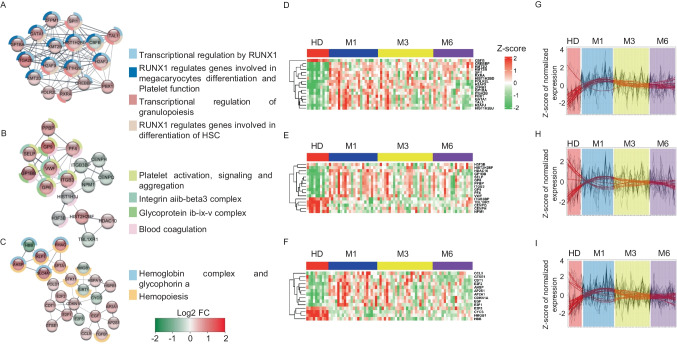
The main molecular complexes associated with the DEGs between convalescent patients at M1 relative to HD and their evolution over the time. **A**–**C** MCODE 1, 2, and 3, respectively, and the main associated pathways. The spheres are colored depending on the gene fold change (Log2FC), as depicted in the figure. Pathways associated with each MCODE are shown as donuts. **D**–**F** MCODE 1, 2, and 3 gene expression level at M1, M3, M6 of convalescence and in HD. **G**–**I** Trend of MCODE 1, 2, and 3 gene expression at M1, M3, M6, and HD

We next performed MCODE analysis using all DEGs observed between COVID-19 patients at M1 and the HD to determine the major mechanisms behind the dysregulation observed in convalescent COVID-19 patients. This approach allowed the identification of three highly connected networks (Fig. 3). Network 1 is distinguished by the upregulation of genes involved in the RUNX1 signaling in the regulation of megakaryocyte and erythroid differentiation, and platelet function (*GATA1*, *SPI1, ZFPM1*, and *TAL1)* [32–35] (Fig. 3A). Importantly, this network included the upregulation of *GP1BA*, a receptor for vWF that leads to platelet adhesion and activation. In parallel, in network 2 (Fig. 3B), we observed the upregulation of several genes involved in platelet pro-coagulation activity and subsequent thrombin and fibrin formation, such as *GP1BB*, *VWF*, *PF4*, *ITGB3*, *GP6*, and *GP9*, which functions as the vWF receptor and mediates vWF-dependent platelet adhesion to blood vessels [36, 37]. Concomitantly, network 3 (Fig. 3C) was enriched for the expression of genes of the hemoglobin complex and glycophorin (*HBB*, *KLF1*, *SLC4A1*, *AHSP*, *RHAG*, and *SPTA1*). Despite the heterogeneity of patients, as illustrated in the heatmaps of individual gene expression (Figs. 3 D–F), these results show an interaction between platelets and neutrophil activation pathways that persists to M3 and M6. Moreover, the dynamics of these changes did not show a return to HD levels at the later follow-up after discharge (Figs. 3 G–I).

In other hand, DEGs (314 genes) shared at M1 and M3 relative to HD (Figure S6A) are mainly involved in protein metabolism (Figure S6B). DEGs specific to convalescent patients at M6 (157 genes) and M3 (50 genes) involved pathways of aminophospholipid translation, heme signaling, cell morphogenesis, and inflammatory response (Figure S6C) and glycerolipid metabolic processes (Figure S6D). We noted significant enrichment of genes involved in erythrocyte differentiation and granulopoiesis (Figure S6E) at M1 and M6 (Figure S6F). These DEGs showed the same kinetics at M1 and M6 (Figure S6G).

### Characterization of a “Core Signature” of Gene Expression in Convalescent COVID-19 Patients

**Fig. 4:**
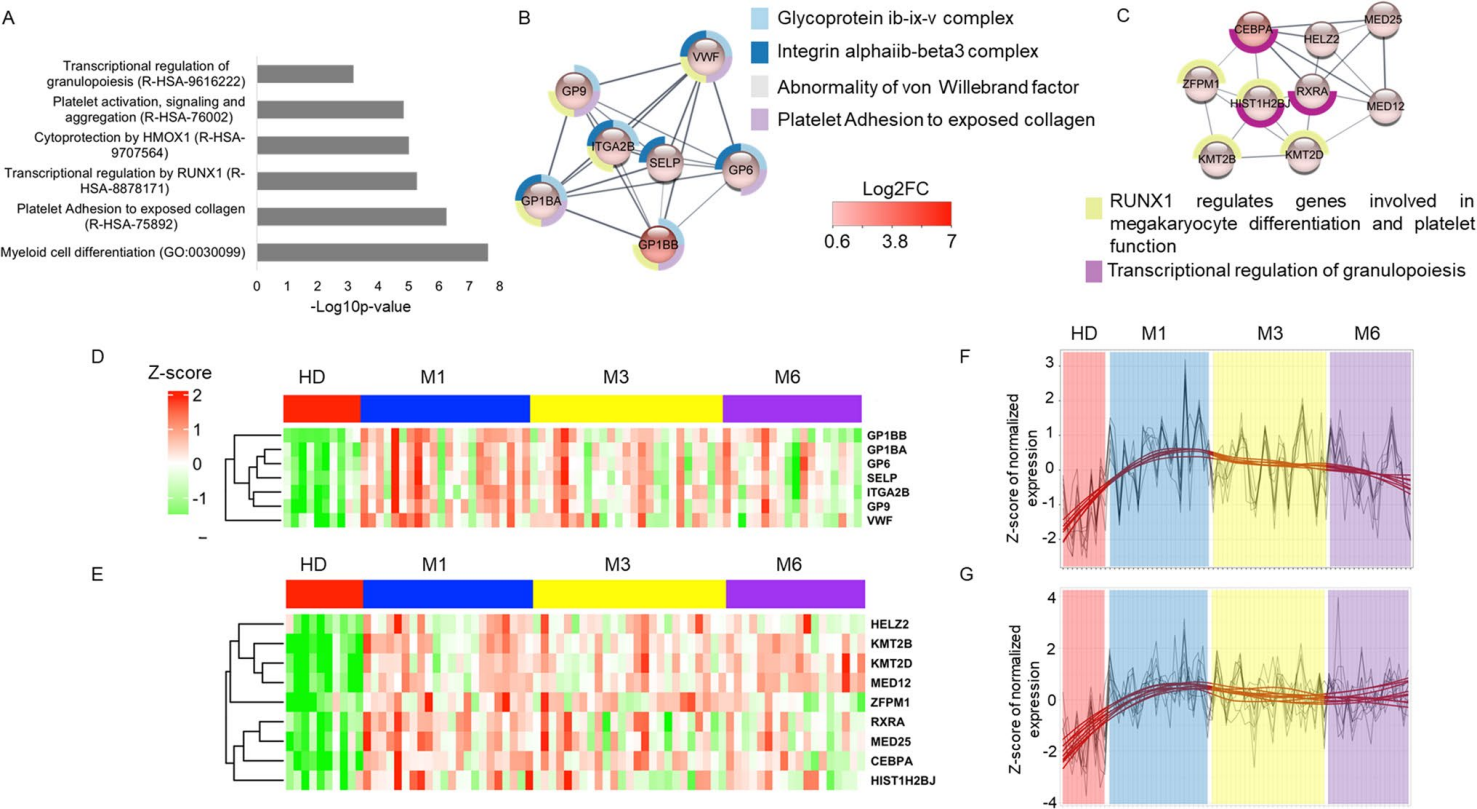
Differentially expressed genes shared between the comparisons of convalescent patients at M1, M3, and M6 to HD. **A** Pathways associated with 477 DEGs shared between convalescent patients at M1, M3, and M6 relative to HD. **B**, **C** MCODE 1 and 2 of the 477 DEGs and their main pathways. The spheres are colored depending on the gene fold change (Log2FC), as depicted in the figure. Pathways associated with each MCODE are shown as donuts. **D**, **E** Heatmap of gene expression belonging to MCODE 1 and 2. **F**, **G** Trend of MCODE 1 and 2 gene expression at M1, M3, M6, and HD

We analyzed pathways involved in the 477 DEGs shared between M1, M3, and M6 relative to HD (Fig. 2A) to identify a “core signature” of convalescent COVID-19 patients throughout the 6 months of follow-up. The main pathways involved are myeloid cell differentiation, platelet adhesion and activation, transcriptional regulation by RUNX1, and granulopoiesis (Fig. 4A). Analysis of the most connected network identified genes that play a critical role in collagen-induced platelet aggregation and thrombus formation (*GP6* and *GP9*) and in the GPIb-V-IX system (*GP1BB*, *GP1BA*). A second cluster consisted of upregulated genes regulated by RUNX1 and involved in megakaryocyte differentiation, platelet function, and the transcriptional regulation of granulopoiesis (*CEBPA* and *ZFPM1*) (Figs. 4B and C). The expression of all these genes was significantly elevated at M1, M3, and M6 of convalescence (Figs. 4D and E) and did not return HD levels by M6 (Figs. 4F and G).

### Gene Expression Profile Associated with Thrombosis in Convalescent Severe COVID-19 Patients

**Fig. 5:**
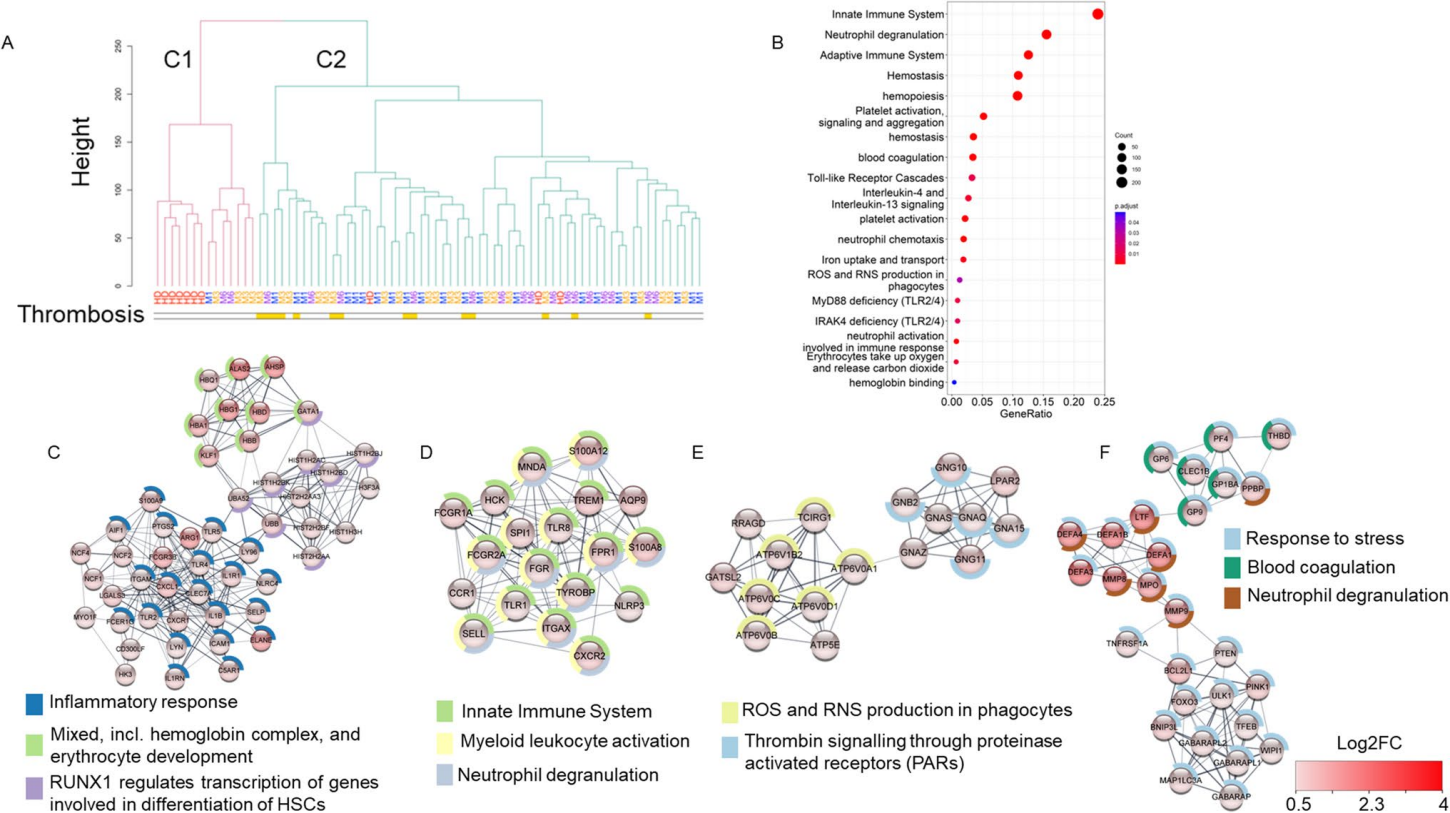
Clustering of HD and convalescent COVID-19 patient samples recovered at M1, M3, and M6 and gene expression profile associated with thrombosis in convalescent COVID-19 patients. **A** Clustering of HD and convalescent COVID-19 patient samples recovered at M1, M3, and M6. **B** Pathways associated with DEGs between convalescent patients who experienced thrombosis in cluster C2 and those who did not experience thrombosis and classified close to HD (convalescent patients in cluster C1). **C**–**F** MCODE 1, 2, 3, and 4 and associated pathways of the DEGs between convalescent patients who experienced thrombosis belonging to cluster C2 and those who did not experience thrombosis classified close to HD (convalescent patients in cluster C1). The spheres are colored depending on the gene fold change (Log2FC), as depicted in the figure. The pathways associated with each MCODE are shown as donuts

We further characterized the gene expression profile that could be associated with thrombosis by performing a clustering analysis of HD and samples from convalescent patients at M1, M3, and M6. The samples were grouped into two main clusters (Fig. 5A). Cluster C1 included HD and convalescent COVID-19 patients who did not experience thrombotic events, showing very similar transcriptome profiles. Cluster C2 included a mixture of convalescent patients who experienced thrombosis and those who did not. This cluster likely corresponds to patients with a high risk of thrombosis. Surprisingly, this cluster included 3 HD who may be at risk of developing thrombosis. DEGs analyses of the convalescent patients in cluster 1 and those who experienced thrombosis in cluster C2 showed 1333 DEGs that were associated with pathways of the innate immune system, neutrophil degranulation, hemopoiesis, platelet activation, and blood coagulation (Fig. 5B). MCODE analysis resulted in four main clusters. The first one (Fig. 5C) included genes involved in neutrophil and complement activation (*S100A9, ARG1, ELANE, C5AR1*, and *FCGR3B*), and in the hemoglobin and erythrocyte development (*ALAS2, GATA2, HBB, HBD, HBG1, HBA1HBQ1*, and *AHSP*). The second cluster (Fig. 5D) corresponded to neutrophil degranulation, with an upregulation of *S100A8, S100A12, SPI1, TYROBP, FGR, FCGR1A*, and *FCGR2A*. Cluster 3 (Fig. 5E) contained mostly genes involved in reactive oxygen and nitrogen species and thrombin signaling. The last cluster (Fig. 5F) contained upregulated genes belonging to the stress response, mainly mediated by neutrophils (*DEFA4, DEFA1B, DEFA3, DEFA1, MPO, LTF, MMP8*, and *MMP9*) and to coagulation (*GP6, GP9, GP1BA, PF4, CLEC1B*, and *THBD*). Overall, convalescent individuals, even those who were asymptomatic, showed persistent changes in the expression of genes involved in inflammation and a status of being prone to thrombosis that lasted for up to 6 months following acute disease.

## Discussion

Here, we report persistent severe abnormalities in the blood of convalescent COVID-19 patients. An abnormal immune cell phenotype, elevated serum markers of inflammation, endothelial activation, and intestinal translocation coexist with anti-inflammatory and epithelial repair markers. Whole blood gene expression analyses showed marked changes in the expression of genes involved in several pathogenic pathways involving hematopoiesis, platelet, erythrocyte, and neutrophil activation. The altered gene expression did not return to the profile of HD after 6 months of follow-up. Another original finding from our study is the identification of a “core signature” of convalescent COVID-19 patients that is conserved for up to 6 months after hospital discharge, suggesting the persistence of pathophysiological processes, not only in patients who are still symptomatic but also those without any “overt” sequelae. Finally, we identified a gene expression profile associated with thrombosis that occurred in a subgroup of convalescent COVID-19 patients.

We first confirmed a decrease in the frequency of peripheral T lymphocytes and a relative increase in the frequency of central memory CD4^+^ and activated effector CD8^+^ T cells until M3 [38]. Concomitantly, serum cytokine profiling showed a balance between pro-inflammatory factors and anti-inflammatory cytokines at M1 and M3. Remarkably, the levels of markers of monocyte/macrophage and endothelial activation, chemotaxis and hematopoietic cytokines, were still elevated several months after acute infection.

Longitudinal follow-up showed changes in blood gene expression, with marked dysregulation of the activation, signaling, and aggregation of platelet homeostasis, transcriptional regulation of granulopoiesis, and neutrophil degranulation. Overall, dysregulation of these pathways was common throughout the convalescent COVID-19 patient population, emphasizing a “core signature,” with upregulation of the RUNX1 pathway, which regulates the expression of many dysregulated genes [33, 39]. Of note, up to 78% of these genes exhibit binding sites for this factor, highlighting the important role of RUNX1 in the physiopathology of COVID-19 [40].

Clinically, the persistence of pathophysiological processes is illustrated by “emergency hematopoiesis” well beyond clinical recovery, as illustrated by the upregulation of key genes involved in the hematopoietic cell differentiation of granulopoiesis, erythroid and megakaryocytic cell, coagulation, platelet activation, and aggregation. These results extend previous observations of abnormal hematopoiesis during the convalescent stage of severe and acute COVID-19 patients [12, 41].

Coagulopathy is an important cause of morbidity and mortality among patients with COVID-19 [42, 43] and SARS-CoV-2 induces endothelial disruption and vascular thrombosis in histopathologic sections of lungs from autopsies of both humans and rhesus macaques infected with SARS-CoV-2 [44]. Histological analysis of pulmonary vessels in acute COVID-19 has shown widespread thrombosis with microangiopathy [45, 46]. Relative to healthy controls, we show persistent upregulation of genes involved in platelet pro-coagulation activity and subsequent thrombin and fibrin formation, as well as genes that mediate vWF-dependent platelet adhesion to blood vessels. Consistent with these observations, a potential role of vWF and complement activation in COVID-19-associated coagulopathy has been proposed [47]. Our results provide a pathophysiological explanation for the higher incident risk of deep vein thrombosis, pulmonary embolism, bleeding, and ischemic cardiovascular events among convalescent COVID-19 patients [48]. Interestingly, a high relative incidence of vascular events was observed soon after COVID-19 diagnosis that declined rapidly but incidence remains elevated up to 7 months after COVID-19 diagnosis [49]. Our study extended this observation by confirming a persistent risk of vascular events and by providing a pathophysiological profile of the convalescent patients. These observations raise the question of the maintenance of prevention measures to avoid thromboembolism events [50].

Elevated NET markers have been reported in the serum of acute severe COVID-19 patients, associated with inflammatory cytokine release, coagulopathy, and respiratory failure [51]. Here, we demonstrate that 6 months after hospital discharge, convalescent patients still exhibit a significant increase in the expression of genes of neutrophil degranulation, NET and alarmins [52], illustrating the role of neutrophils in blood coagulation and thrombosis, probably via interaction with both the injured endothelium and fibrinogen [53–55]. Overall, our data underscore the central role of the trio of platelets, neutrophils, and erythrocytes in the disturbances observed in convalescent severe COVID-19 patients.

We sought to characterize the gene expression profile associated with thrombotic events by clustering analysis. Despite a degree of heterogeneity, all convalescent patients who experienced thrombosis were grouped within the same cluster. This cluster was highly dominated by dysregulation of the RUNX1 TF, neutrophil degranulation, and thrombin signaling pathways. Convalescent COVID-19 patients who experienced thrombosis also showed upregulation of hemoglobin complex and erythrocyte development genes, as well as genes of platelet activation and blood coagulation. It is now admitted that infections increase the risk of thrombosis independently of the risk factors for thromboembolic diseases [56]. Beside SARS-CoV-2, influenza and cytomegalovirus can also enhance the risk of thrombosis. The key factor that probably underlies the risk of thrombosis is the level of inflammation induced by the infection, which can activate platelets through different receptors, triggering aggregation and thrombi formation. Interestingly, the hospitalized COVID‐19 patients have significant higher thrombosis incidence relative to hospitalized patients with influenza [57]. This difference is probably due to a higher pro-coagulant status induced in COVID-19 subsequent to the activation of both platelets, neutrophils and erythrocytes as confirmed in our study.

The reasons for the persistence of dysregulation after clinical recovery are not obvious but suggest a continuous pathophysiological process both in patients who are still symptomatic and those without any “overt” clinical sequelae [58–62]. The mechanism behind this phenomenon is still unknown, although several hypotheses have been suggested, such as viral reactivation and/or persistent viral reservoirs or antigens [63, 64]. In addition, we show high expression of markers that suggest a lack of integrity in the intestinal barrier and ongoing tissue repair in convalescent COVID-19 patients, suggesting the persistence of a pathological phenomenon at the mucosal level [65].

Our study had several limitations. Because of the limitations in sampling, all assays were not performed in the global cohort and a limited number of patients were sampled at M1, M3, and M6. Furthermore, the convalescent patients were not perfectly matched in age and gender with healthy donors. Indeed, convalescent patients and healthy donors were of 66% and 100% males with mean age of 59 and 35 years, respectively. Another limitation of this study is the possible heterogeneity with regard to the time from initial infection by SARS-Cov-2, the duration of hospitalization, and the severity of the disease. In this study, we followed mostly hospital patients who experienced severe COVID-19. It will be informative to investigate whether certain immune and transcriptomic signatures are reproducibly identified in moderate COVID-19 patients.

In conclusion, we describe a complex pathophysiological process in which both severe mediators of tissue injury and thrombotic complications and mechanisms for the control of a hyperinflammatory state coexist in patients who are still symptomatic (50% of our cohort) and those without overt symptoms. Our results suggest the persistence of activation of neutrophils and disturbance of the coagulation pathway in many convalescent COVID-19 patients. Hence, convalescent sever COVID-19 patients who experienced thrombotic events during the acute phase of the disease show an upregulation of genes involved in neutrophil activation, platelet, and blood coagulation. The lack of restoration of gene expression to a normal profile after up to 6 months of follow-up indicates the need to carefully extend their clinical follow-up and propose preventive measures.

## The French COVID cohort study group

Laurent ABEL^11^, Amal ABROUS^12^, Claire ANDREJAK^13^, François ANGOULVANT^14^, Delphine BACHELET^15^, Marie BARTOLI^16^, Sylvie BEHILILL^17^, Marine BELUZE^18^, Krishna BHAVSAR^15^, Anissa CHAIR^15^, Charlotte CHARPENTIER^15^, Léo CHENARD^15^, Catherine CHIROUZE^19^, Sandrine COUFFIN-CADIERGUES^12^, Camille COUFFIGNAL^15^, Nathalie DE CASTRO^20^, Marie-Pierre DEBRAY^15^, Dominique DEPLANQUE^21^, Diane DESCAMPS^15^, Alpha DIALLO^16^, Fernanda DIAS DA SILVA^12^, Céline DORIVAL^22^, Xavier DUVAL^15^, Philippine ELOY^15^, Vincent ENOUF^17^, Hélène ESPEROU^12^, Marina ESPOSITO-FARESE^15^, Manuel ETIENNE^23^, Aline-Marie FLORENCE^15^, Alexandre GAYMARD^24^, Tristan GIGANTE^25^, Morgane GILG^25^, François GOEHRINGER^26^, Jérémie GUEDJ^27^, Ikram HOUAS^12^, Isabelle HOFFMANN^15^, Jean-Sébastien HULOT^28^, Salma JAAFOURA^12^, Simon JAMARD^29^, Ouifiya KAFIF^15^, Antoine KHALIL^15^, Nadhem LAFHEJ^15^, Samira LARIBI^15^, Minh LE^15^, Quentin LE HINGRAT^15^, Soizic LE MESTRE^16^, Sophie LETROU^15^, Bruno LINA^24^, Guillaume LINGAS^30^, Denis MALVY^31^, France MENTRÉ^15^, Hugo MOUQUET^17^, Nadège NEANT^17^, Christelle PAUL^16^, Aurélie PAPADOPOULOS^12^, Ventzislava PETROV-SANCHEZ^16^, Gilles PEYTAVIN^15^, Valentine PIQUARD^15^, Olivier PICONE^32^, Manuel ROSA-CALATRAVA^24^, Bénédicte ROSSIGNOL^25^, Patrick ROSSIGNOL^26^, Carine ROY^15^, Marion SCHNEIDER^15^, Coralie TARDIVON^15^, Jean-François TIMSIT^15^, Sarah TUBIANA^15^, Sylvie VAN DER WERF^17^, Benoit VISSEAUX.^15^

^11^ Inserm UMR 1163, Paris, France.

^12^ Inserm sponsor, Paris, France.

^13^ Service de pneumologie et réanimation respiratoire, CHU Amiens, France.

^14^ Hôpital Necker, Paris, France.

^15^ Hôpital Bichat, Paris, France.

^16^ ANRS-MIE, Paris, France.

^17^ Pasteur Institute, Paris, France.

^18^ F-CRIN Partners Platform, Paris, France.

^19^ CHRU Jean Minjoz, Besançon, France.

^20^ Hôpital Saint Louis, Paris, France.

^21^ Hôpital Calmette, Lille, France.

^22^ Inserm UMR 1136, Paris, France.

^23^ CHU Rouen, France.

^24^ Inserm UMR 1111, Lyon, France.

^25^ F-CRIN INI-CRCT, Nancy, France.

^26^ CHU Nancy, France.

^27^ Inserm UMR 1137, Paris, France.

^28^ Hôpital Européen Georges Pompidou, Paris, France.

^29^ Hôpital Bretonneau, Tours, France.

^30^ Inserm UMR 1137, Paris, France.

^31^ CHU Bordeaux, France.

^32^ Hôpital Louis Mourier, Colombes, France.

## Supplementary Information

Below is the link to the electronic supplementary material.

Supplementary file1 (DOCX 3597 KB)

## Data Availability

RNA-sequencing data that support the findings of this study have been deposited in the Gene Expression Omnibus (GEO) repository with the accession codes GSE227116. The hg19 human reference genome can be found in the NCBI database.
