# Correction to: Neutrophil Activation and Immune Thrombosis Profiles Persist in Convalescent COVID‑19

**DOI:** 10.1007/s10875-023-01477-9

**Published:** 2023-03-30

**Authors:** Hakim Hocini, Aurélie Wiedemann, Fabiola Blengio, Cécile Lefebvre, Minerva Cervantes‑Gonzalez, Emile Foucat, Pascaline Tisserand, Mathieu Surenaud, Séverin Coléon, Mélanie Prague, Lydia Guillaumat, Corinne Krief, Craig Fenwick, Cédric Laouénan, Lila Bouadma, Jade Ghosn, Giuseppe Pantaleo, Rodolphe Thiébaut, the French COVID cohort study group, Yves Lévy

**Affiliations:** 1 Vaccine Research Institute, Université Paris-Est Créteil, Faculté de Médecine, INSERM U955, Team 16, Créteil, France; 2 Department of Public Health, Univ. Bordeaux, Inserm Bordeaux Population Health Research Centre, Inria SISTM, UMR 1219, Bordeaux, France; 3 Service of Immunology and Allergy, Department of Medicine, Lausanne University Hospital and University of Lausanne, Lausanne, Switzerland; 4 Département Épidémiologie Biostatistiques Et Recherche Clinique, AP-HP, Hôpital Bichat, INSERM, Centre d’Investigation Clinique‑Epidemiologie Clinique 1425, 75018 Paris, France; 5 UMR 1137, Université de Paris, INSERM, IAME, 75018 Paris, France; 6 APHP- Hôpital Bichat – Médecine Intensive et Réanimation des Maladies Infectieuses, Paris, France; 7 AP-HP, Hôpital Bichat, Service de Maladies Infectieuses Et Tropicales, 75018 Paris, France; 8 Swiss Vaccine Research Institute, Lausanne University Hospital, University of Lausanne, Lausanne, Switzerland; 9 CHU de Bordeaux, Pôle de Santé Publique, Service d’Information Médicale, Bordeaux, France; 10 Assistance Publique‑Hopitaux de Paris, Service Immunologie Clinique, Groupe Henri-Mondor Albert-Chenevier, Créteil, France

**Correction to** **:** **Journal of Clinical Immunology**

**https://doi.org/10.1007/s10875-023-01459-x**

Due to typesetting mistake, the institutional authors of the French COVID cohort study group, were mistakenly listed in the author group which resulted to redundancy. Also, Yves Lévy should be the last author from the list as shown above.

The original version has been corrected.

