# Supplementary material for: Neutrophil Activation and Immune Thrombosis Profiles Persist in Convalescent COVID-19
Source: J Clin Immunol. 2023 Mar 21;43(5):882–93. doi: 10.1007/s10875-023-01459-x (PMC10029801; doi:10.1007/s10875-023-01459-x)

**Supplementary Information**

Hocini. H *et al*.

Contributor Information

Yves Lévy: Email: [yves.levy@aphp.fr](mailto:yves.levy@aphp.fr)

**Materials and Methods**

**Cell phenotyping**

Immune phenotyping was performed using an LSR Fortessa 4-laser (488, 640, 561, and 405 nm) flow cytometer (BD Biosciences) and FlowJo software version 9.9.6 (Tree Star Inc.). CD4^+^ and CD8^+^ T cells were analyzed for CD45RA and CCR7 expression to identify the naive, memory, and effector cell subsets and for co-expression of the activation markers HLA-DR and CD38.

**Antibody titration and pseudo-neutralization assay**

Anti-Spike IgG from sera of the donors were titrated by multiplex bead assay. Briefly, Luminex® beads were coupled to SARS-CoV-2 Spike antigens as previously described (Fenwick, Croxatto, *et al.*, 2021) and added to a Bio-Plex plate (Bio-Rad). Beads were washed using a magnetic plate washer (Bio-Rad) and incubated with serially diluted individual serum samples. Binding was assessed using an anti-human IgG-PE secondary antibody (One Lambda, #LS-AB2). Fluorescence was read directly on a Bio-plex 200 plate reader (Bio-Rad). Binding assays were further completed by a SARS-CoV-2 Spike / ACE-2 Cell-Free Surrogate Neutralization Assay following a previously established protocol (Fenwick, Turelli, *et al.*, 2021). Briefly, an ACE-2 mouse Fc fusion protein (Creative Biomart) was added to beads coupled with SARS-CoV-2 Spike and previously incubated with human sera. Median Fluorescence Intensity (MFI) of beads associated with the ACE-2 receptor was measured using a PE F(ab')2-Goat anti-Mouse IgG (H+L) (Thermo Fisher, 12-4010-87). The relative neutralization of serum Ig was calculated using a standard curve with anti-SARSs-CoV-2 RBD Neutralizing Antibody (SAD-S35 Acro-Biosystems), with 10 µg/mL corresponding to the MFIMax inhibition and 13.7 pg/mL the MFIMax binding, using the formula: % Inhibition = (100- ([MFITest dilution – MFIMax inhibition] / [MFIMax binding - MFIMax inhibition])).

**Table S1. Demographic and clinical characteristics of the patients.**

| **Demographic characteristics** | **Number of patients** | |  |  |  |  |
| --- | --- | --- | --- | --- | --- | --- |
| Age - Median (IQR) - years | 100 | 59 [47-67] |  |  |  |  |
| Male sex – No./total No. (%) | 100 | 67/100 (67) |  |  |  |  |
| Intensive care unit (ICU) during acute phase | 100 | 81/100 (81) |  |  |  |  |
| **Thrombotic event between D1 and D15 during the acute phase** | 10 |  |  |  |  |  |
| **Persistent symptoms after hospital admission** | **M1 (n=64)** | **Missing** | **M3 (n=53)** | **Missing** | **M6 (n=20)** | **Missing** |
| *Cough* | 19 | 11 | 6 | 6 | 5 | 0 |
| *With sputum production* | 6 | 11 | 5 | 6 | 0 | 0 |
| *Dyspnea* | 14 | 11 | 15 | 6 | 8 | 0 |
| *Sore throat* | 1 | 11 | 2 | 6 | 1 | 0 |
| *Rhinorrhea* | 2 | 11 | 5 | 6 | 1 | 0 |
| *Myalgia* | 3 | 11 | 6 | 6 | 8 | 0 |
| *Arthralgia* | 2 | 11 | 4 | 6 | 4 | 0 |
| *Fatigue* | 26 | 11 | 23 | 6 | 12 | 0 |
| *Headache* | 6 | 11 | 5 | 6 | 3 | 0 |
| *Anosmia* | 3 | 11 | 5 | 6 | 0 | 0 |
| *Ageusia* | 3 | 11 | 4 | 6 | 1 | 0 |
| *Abnormal pulmonary auscultation* | 12 | 11 | 8 | 6 | 5 | 0 |
| *Crackling lung sounds* | 3 | 11 | 0 | 6 | 0 | 0 |
| *Wheezing* | 0 | 11 | 0 | 6 | 1 | 0 |
| *Signs of right heart failure* | 0 | 11 | 1 | 6 | 0 | 0 |
| *Hepatomegaly* | 0 | 11 | 1 | 6 | 0 | 0 |
| *Adenopathy* | 2 | 11 | 0 | 6 | 1 | 0 |
| *Other clinical signs* | 4 | 11 | 6 | 6 | 3 | 0 |
| **No symptoms (%)** | 15 (28) |  | 11 (23) |  | 0 (0) |  |
| **At least one symptom (%)** | 37 (70) |  | 36 (76) |  | 20 (100) |  |
| **Three or more symptoms (%)** | 19 (36) |  | 13 (28) |  | 9 (45) |  |

**Table S2. Details of blood samples used in each assay.**

| **Blood collection** |  |
| --- | --- |
| Multiple time points, n | **33** |
| 2 | 31 |
| 3 | 2 |
| Single time point, n | **69** |
| **Assays performed in the study** |  |
| Phenotype analysis | 66 samples (n = 42 patients) |
| Anti-S IgG and Neutralization | 119 samples (n = 93 patients) |
| Biomarker expression in serum | 115 samples (n = 91patients) |
| Transcriptomic analysis | 65 samples (n = 46 patients) |
|  |  |

| **Assay** | **T cell  phenotype** | **Serum cytokines  measurement** | **RNA-seq** |
| --- | --- | --- | --- |
| **n** | 15 | 30 | 10 |
| **Age-years-Median (IQR)** | 35 (28-42) | 27.5 (23-51) | 33 (29-38) |
| **Male sex-no/total no (%)** | 15/15 (100) | 12/15 (80) | 10/10 (100) |

**Table S3. Characteristics of healthy donors (HDs) involved in the various assays.**

**Figure S1. Quantification of serum-soluble mediators differentially expressed in HDs and convalescent COVID-19 patients at M1, M3, and M6 post-infection**. Measurement of serum-soluble mediators (pg/ml) from n = 30 HDs and n = 42 M1, n= 47 M3, and n=16 M6 with the Bio-Plex 200 System^TM^ (Bio-Rad). A. Pro-inflammatory and anti-inflammatory cytokines. B. Regulatory/Th2 cytokines.

**B**

**A**


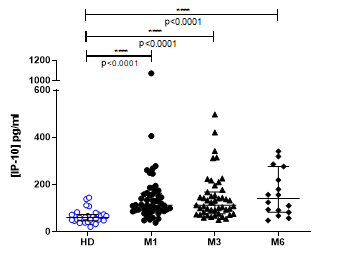

C. Macrophage and endothelial activation markers.

**C**

D. Chemotaxis and hematopoietic cytokines.


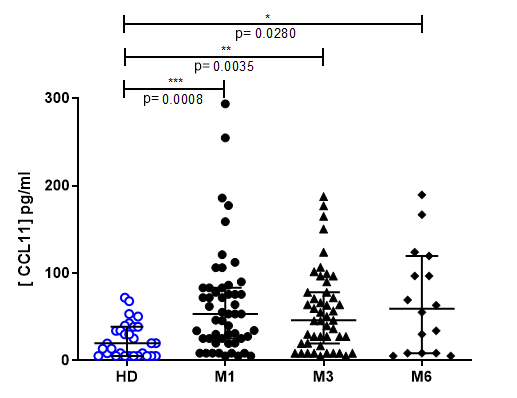


**D**

E. Microbial Translocation in the gut. The differences between HDs and convalescent COVID-19 patients were evaluated using Kruskal Wallis tests. Median values ± IQR are shown.

**E**

**Figure S2. Modifications in immune cell subset frequencies and SARS-CoV-2-specific humoral responses in convalescent COVID-19 patients.** A. Cumulative T-cell subpopulation frequencies and CD8^+^ T-cell activation analyses from n = 15 HD and n = 24 M1, n = 24 M3, and n = 18 M6. B. Anti-Spike immunoglobulin levels and relative neutralization were measured in the sera of convalescent COVID-19 patients at different time points post-infection (n = 57 M1, n = 45 M3, n = 17 M6). For anti-spike IgG, MFI signals for serum antibody binding are expressed as a ratio compared to a negative control pool of healthy pre-COVID-19 pandemic adults (n = 5 HD) (Fold Increase). The frequency of relative neutralization was calculated using an anti-RBD neutralizing antibody in a standard curve. Median values ± IQR are shown and Kruskal Wallis tests were used for comparisons.

**Gated on CD3+CD8+**

**Gated on CD3+CD4+**

**Gated on live PBMCs**

**A**

**B**

**Figure S3. Dynamics of the expression of myeloid-associated genes in the comparison between convalescent COVID-19 patients at M1 and HDs.** A. Heatmap of gene expression of convalescent patients at M1, M3, and M6 and HDs. B. Trend of myeloid-related gene expression profiles. C. Protein-protein interaction (PPI) network and main associated pathways.


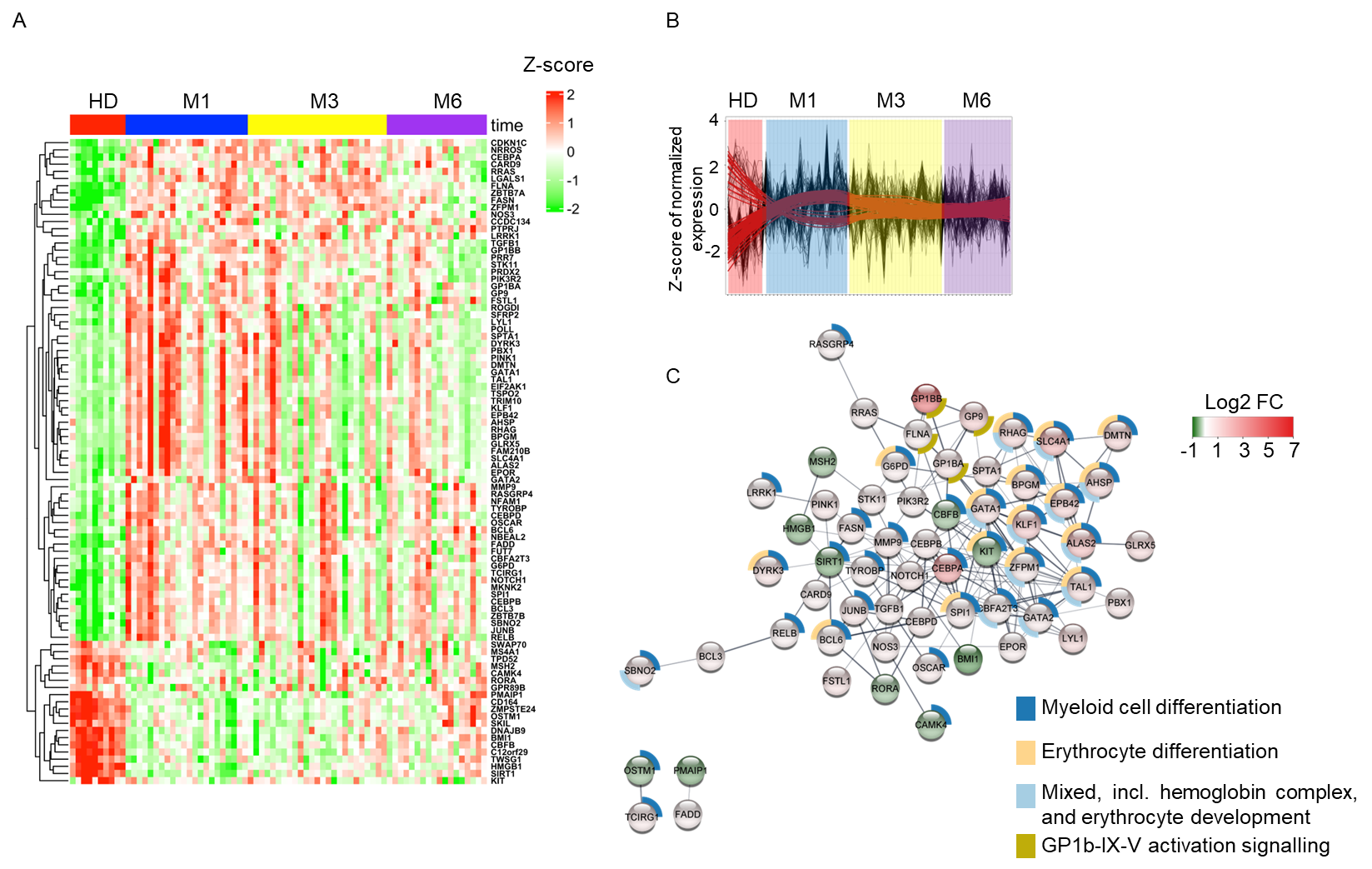


**Figure S4. Dynamics of the expression of platelet-associated genes in the comparison between convalescent COVID-19 patients at M1 and HDs. A.** Heatmap of gene expression of convalescent patients at M1, M3, and M6 and HD. **B.** Trend of platelet-related gene expression profiles. **C.** Protein-protein interaction (PPI) network and main associated pathways.

**
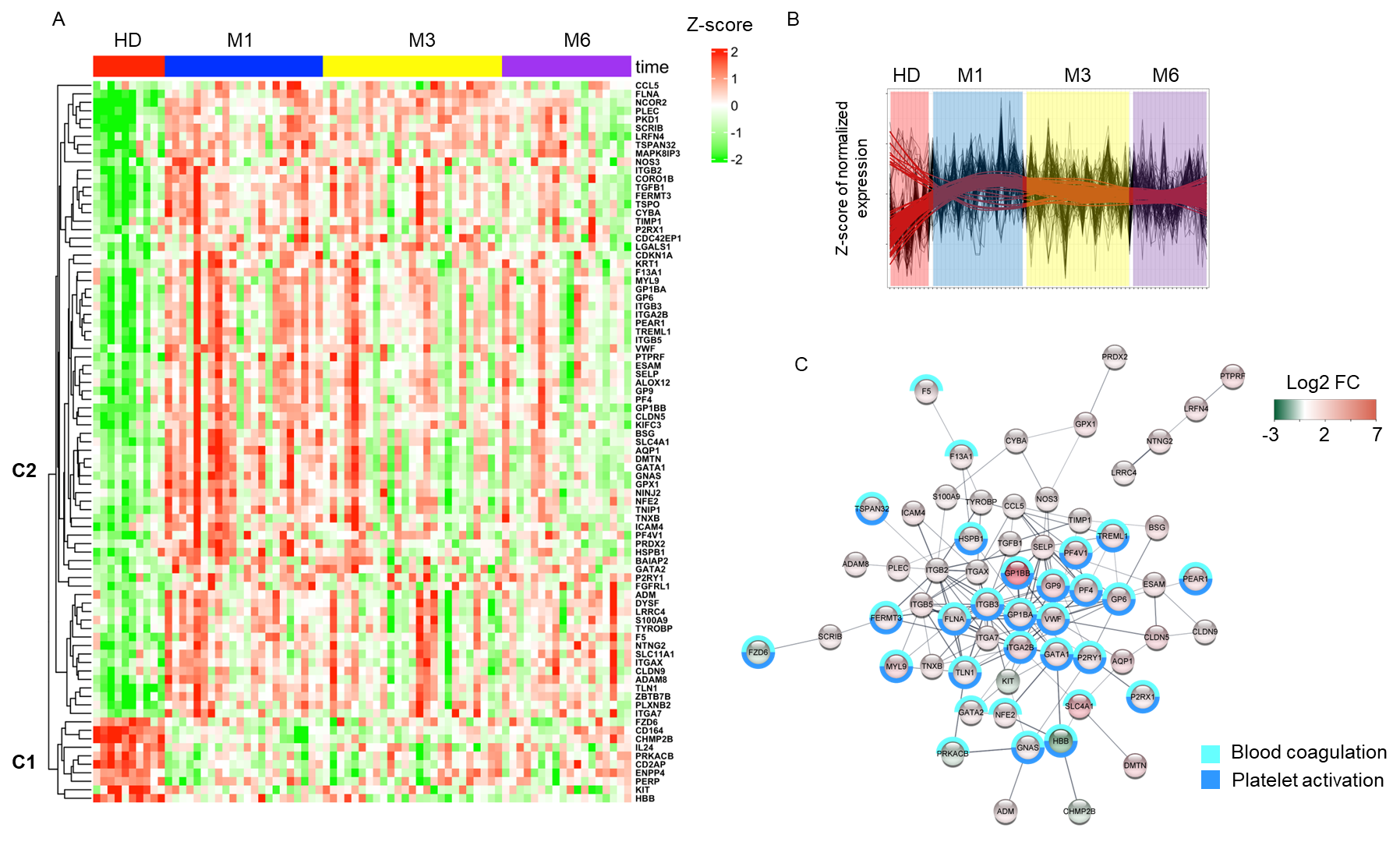
**

**Figure S5. Dynamics of the expression of neutrophil-associated genes in the comparison between convalescent COVID-19 patients at M1 and HDs.** A. Heatmap of gene expression of convalescent patients at M1, M3, and M6 and HD. B. Trend of neutrophil-related gene expression profiles. C. Protein-protein interactions (PPIs) network.

**
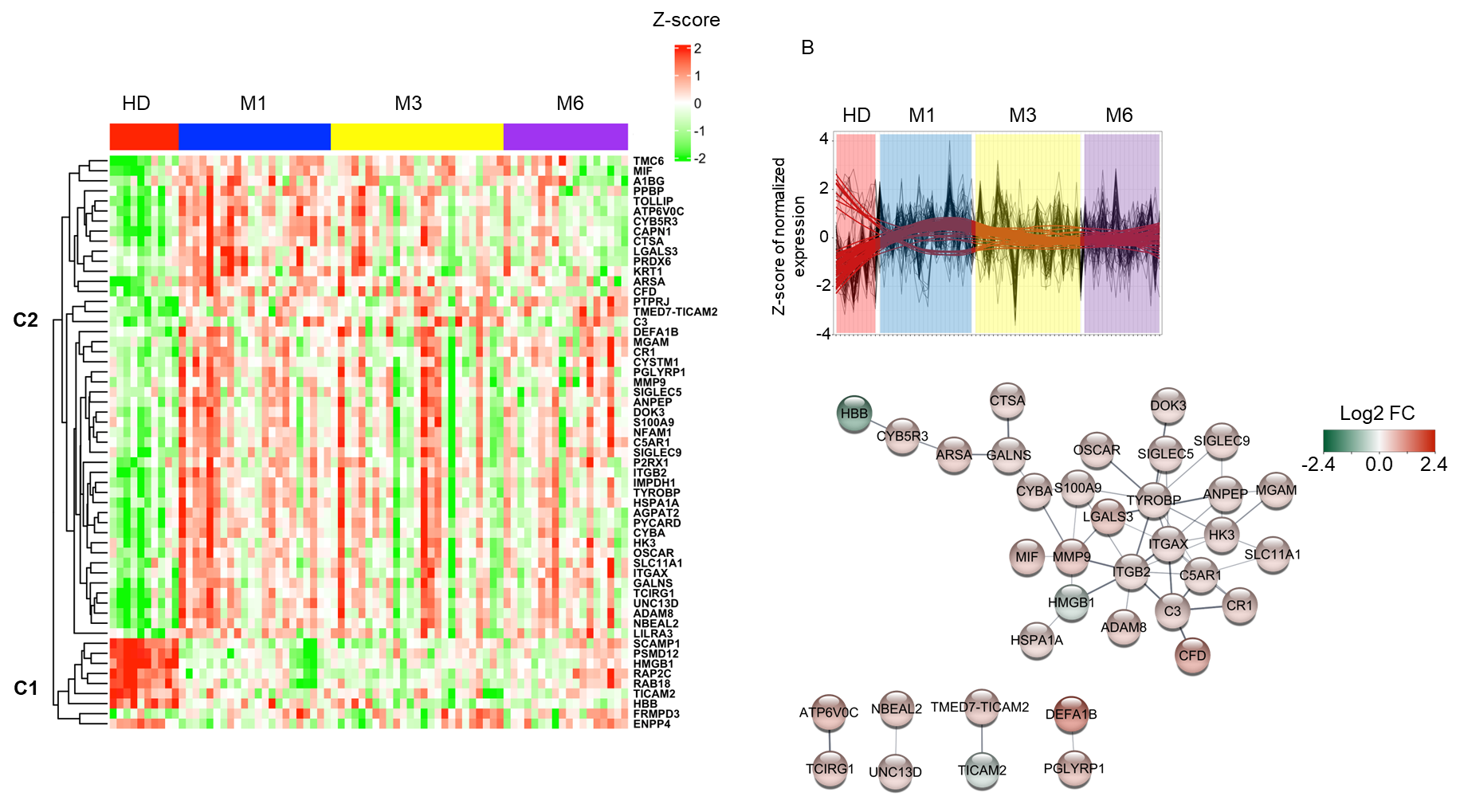
**

C

A

**Figure S6. Pathways associated with differentially expressed genes of convalescent patients at M1, M3, and M6 relative to HDs**. **A.** Venn diagram of the number of DEGs between convalescent severe COVID-19 patients at M1, M3, and M6 relative to HDs. **B.** Pathways associated with the 314 DEGs shared between the comparisons of convalescent patients at M1 and M3 to HDs. **C.** Pathways associated with the 157 DEGs specific to the comparison of convalescent patients at M6 to HD. **D.** Pathways associated with the 50 DEGs specific to the comparison of convalescent patients at M3 to HD. E**.** Pathways associated with the 214 DEGs shared between the comparisons of convalescent patients at M1 and M6 to HD. F. Protein-protein interaction (PPI) network and main associated pathways associated with the 214 DEGs. G. Fold change of the PPI genes associated with the 214 DEGs.


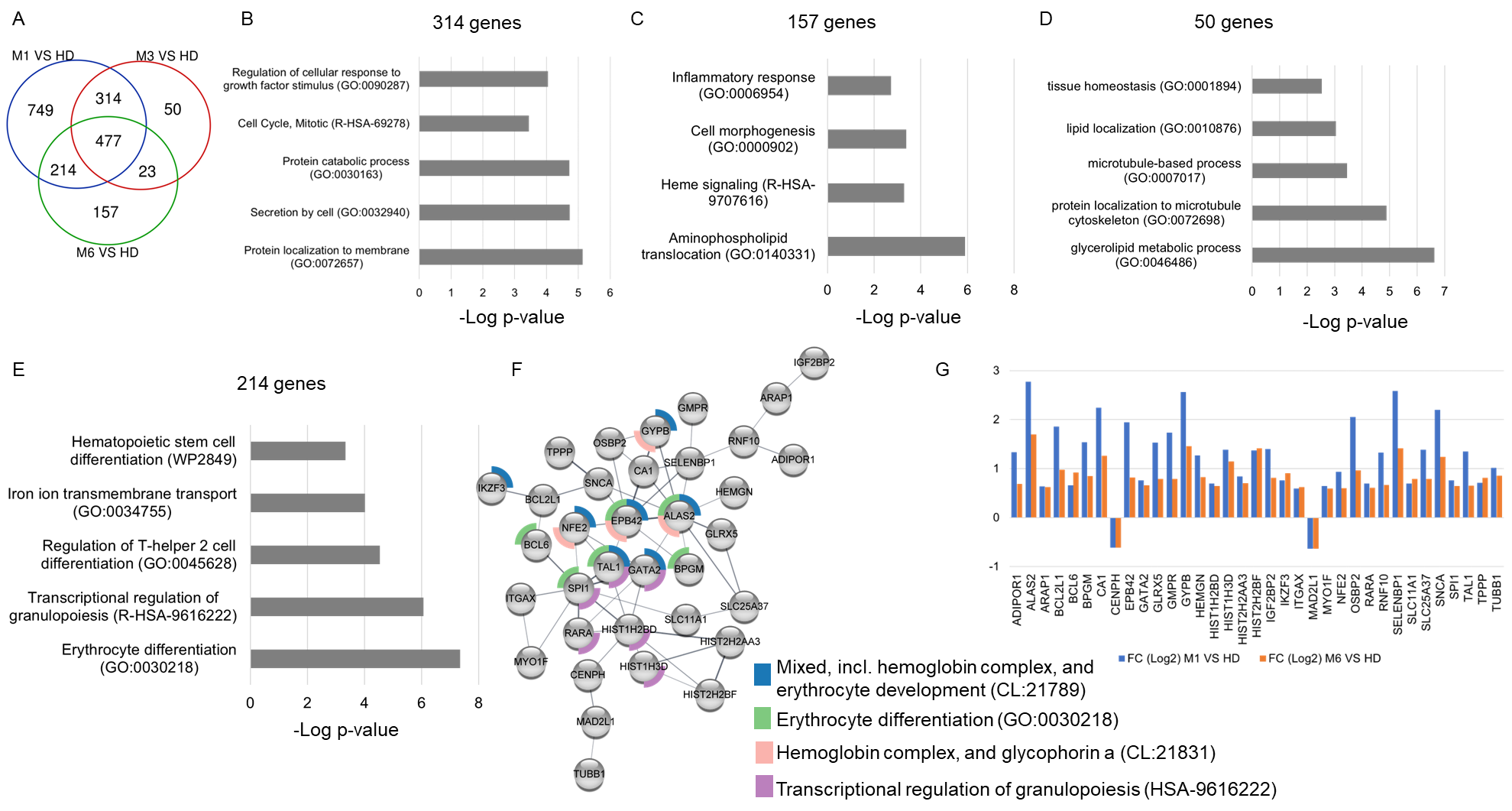


**Figure S7. Selection of the best algorithm for cluster analysis and the best number of clusters.** **A.** The optimal score table obtained with the clValid R package returned hierarchical clustering as the optimal validation measure, along with ”2” as the optimal number of clusters. **B.** The optimal number of clusters was confirmed using the “average silhouette width” score from the factoextra R package.

A. B.


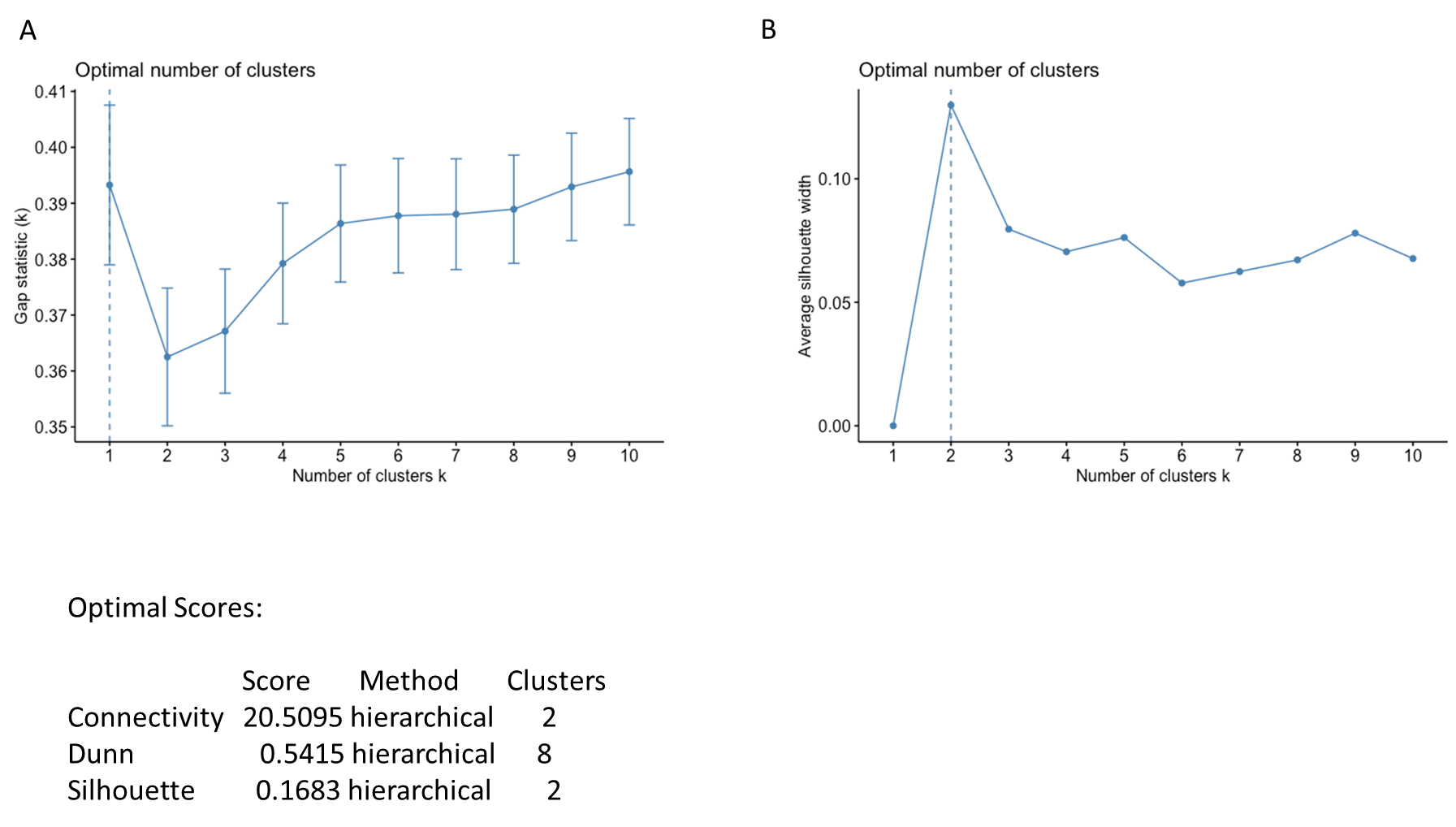

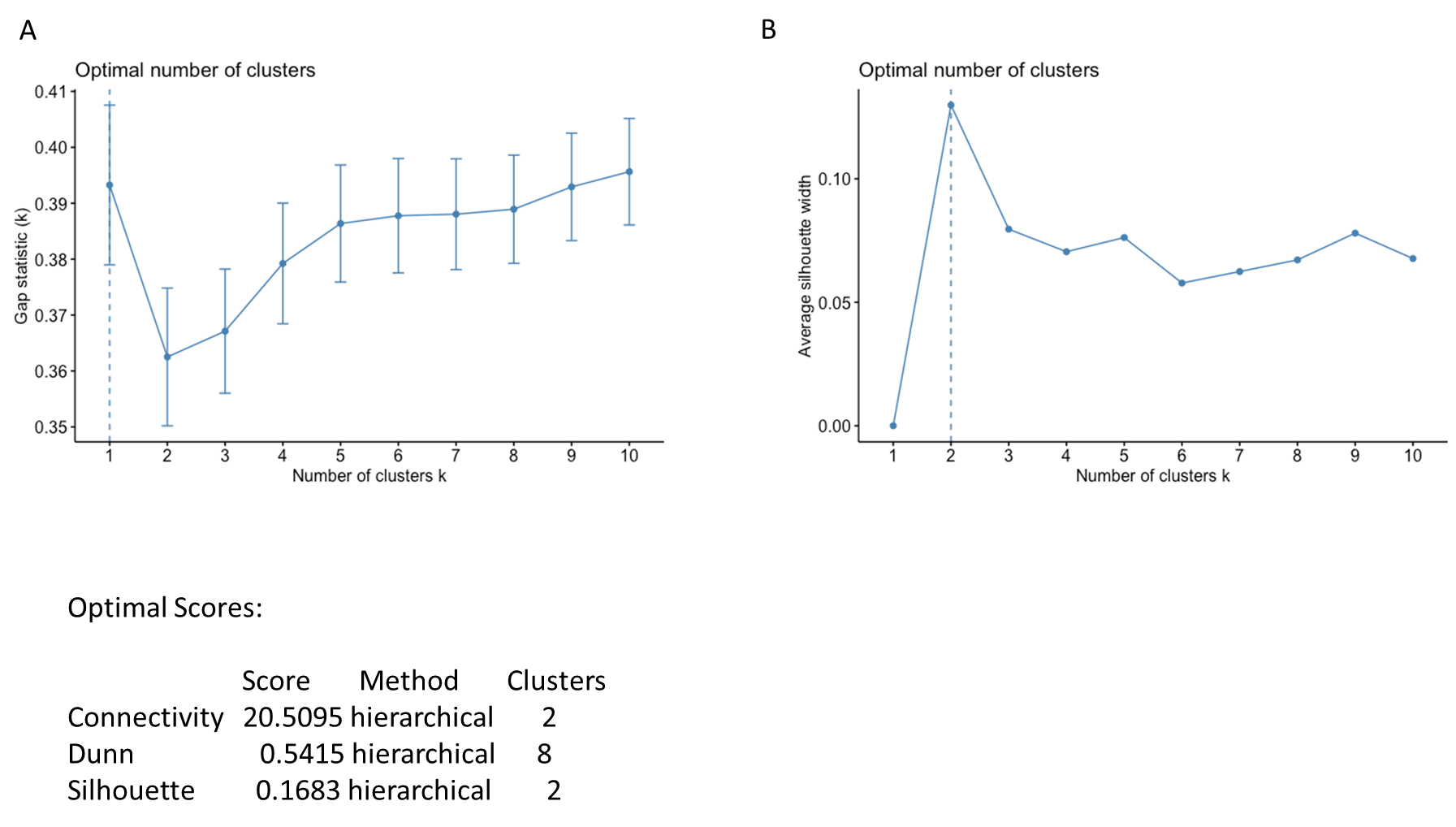

Supplement: Supplementary file 1 — Supplementary file1 (DOCX 3597 KB) [file 10875_2023_1459_MOESM1_ESM.docx]
